# Evaluation of Selected Outcomes of Combination Antiretroviral Therapy: Yemen Cohort Retrospective Descriptive Studies

**DOI:** 10.1038/s41598-019-56314-0

**Published:** 2019-12-27

**Authors:** Mayada Faisal Nabih, Sharifa Ezat Wan Puteh, Amrizal Muhammad Nur

**Affiliations:** 1 National AIDS Control Program – Ministry of Public Health and Population, Sana’a, Yemen; 2 Community Health Department - Faculty of Medicine Universiti Kebangsaan Malaysia (UKM), Kuala Lumpur, Malaysia; 3 International Centre for Casemix and Clinical Coding (ITCC) - Faculty of Medicine Universiti Kebangsaan Malaysia (UKM), Kuala Lumpur, Malaysia

**Keywords:** Drug delivery, Outcomes research

## Abstract

In 2007, HIV treatment services were established in five main governorates out of twenty-two which resulted in low access to services and poor treatment outcomes. The main goal of this study was to evaluate and analyse the selected treatment outcomes of eight cohorts of PLHIV who were treated with cART during 2007–2014. The method used was a retrospective descriptive study of 1,703 PLHIV who initiated cART at five public health facilities. The results: Retention rate was less than 80%, male: female ratio 1.661, with a mean age of 35 years (±9.2 SD), 85% had been infected with HIV via heterosexual contact. 65% of patients presented with clinical stages 3 and 4, and 52% of them were initiated cART at a CD4 T-cell count ≤200 cells/mm. 61% of cART included Tenofovir and Efavirenz. TB treatment started for 5% of PLHIV, and 22% developed HIV-related clinical manifestations after cART initiation. 67% of PLHIV had experienced cART substitution. The mean AIDS-mortality rate was 15% and the mean LTFU rate was 16%. Conclusion: Although cART showed effectiveness in public health, mobilization of resources and formulation of better health policies are important steps toward improving access to cART and achieving the desired treatment outcomes.

## Introduction

Combination prevention strategies are considered the current focus of public health efforts to reduce the spread of human immunodeficiency virus (HIV)^1^. Early initiation of combination antiretroviral therapy (cART) suppresses viral replication, reduces morbidity and mortality among people living with HIV (PLHIV) and reduces the probability of HIV transmission^2,3^. A test and treatment cascade is recommended to all PLHIV regardless of CD4 cell count or WHO clinical stages^4,5^.

Female sex workers (FSWs), men who have sex with men (MSM) and injecting drug users (IDUs) are considered as key drivers of the HIV epidemic^6^. In 2010, the population size estimate among FSW and MSM showed that there were 60000 FSW and 44000 MSM in Yemen, and the highest number was among the age group of 20–29 and 15–34 years respectively. However, homosexuality and extramarital sex are forbidden in Yemen as it is criminalized by law. Therefore, lesbians, gays and transgender are pressured to hide their sexual identity, and they are less likely to seek HIV services^7^. In 2008, the seroprevalence study among FSW in Yemen showed HIV prevalence 1.3%, while HIV prevalence among MSM was around 6% as shown in the study that was conducted in 2011^8^.

CD4 cell count is the immunological test that was previously used as the main test to initiate cART and as a baseline for all PLHIV who are enrolled in care. It identifies PLHIV who have advanced disease with a CD4 cell count ≤200 cell/mm3. It is also considered the best predictor for disease status and immediate risk of death^9,10^, but it is a poor predictor for treatment failure^11^. Viral suppression and viral failure are monitored by viral load (VL) test which is an important test for all people on cART^10,12^. However, both tests (CD4 cell count and VL tests) are not sustainably available in Yemen, because of its supply is coming from donors support^13^.

The WHO clinical stages for HIV disease can also identify disease progression which ranges from stage one (asymptomatic) to stage four (advanced acquired immune deficiency syndrome (AIDS))^10,12^. Most identified HIV cases in Yemen are advanced AIDS. Tuberculosis (TB) is a common co-infection and a leading cause of morbidity and mortality among PLHIV globally including Yemen^13^. In 2014, there were 1.2 million infected with TB and 390000 deaths. TB risk increases with progressive immunodeficiency. However, it can occur at any CD4 cell count. But cART plays a key role in decreasing TB infection among PLHIV and reducing the fatality rate^14^.

Retention in HIV care has been defined as the time where PLHIV go through from the date of HIV diagnosis and link to HIV prevention, treatment, and support services and the continuation of lifelong ART. This lifelong cART care leads to reduced morbidity and mortality, reduced new HIV infection among children and adult and reduced treatment failure^15^. Very high levels of treatment coverage and viral suppression are required to reduce HIV transmission in the community. However, achieving 80% treatment coverage is important to reduce HIV incidence in the community^16^.

The first case of HIV disease in Yemen was described in 1987 and the overall HIV prevalence among the general population is low at 0.2%, in a population of approximately 30 million people. Although published data of December 2014 put the estimated number of persons infected with HIV at 35,000, it is now estimated that about 9194 PLHIV in Yemen by using the Spectrum software that was developed by the UNAIDS. This software created new projections for key indicators by using demographic data and program, HIV surveillance and survey. The magnitude of the problem has been recognized and is receiving the highest political commitment. In a similar effort, the National AIDS Control Program/Ministry of Public Health and Population (NAP/MOPHP) with the WHO conducted a need assessment for scaling up comprehensive HIV care to five ART operational sites at five public hospitals. The five sites are located in five governorates out of 22 governorates. A recent report indicates that there have been 4231 newly registered HIV cases since 1987. Most of these cases affect the ages of 25 to 49 years and of whom, 1703 are receiving treatment and care^13,17,18^.

Most PLHIV are lost to follow-up (LTFU) at a pre-ART period which considered a major cause of poor HIV care program performance^17^. The recent WHO new recommendations of 2016 for early initiation of cART help facilitate the identification of newly diagnosed patients and minimize the LTFU^10^. Most of the global challenges are related to patient attrition, contributing to suboptimal treatment outcomes due to delayed cART initiation and as a result high morbidity and mortality^10,19^. Early initiation of cART remains a concern as most PLHIV who are not well prepared to start cART may not be able to adhere to treatment well and may lead to adverse consequences on treatment outcomes. However, early initiation of cART as recommended by the WHO will promote immune restoration and prevent HIV transmission^10,20^.

Limited resources, stigma and discrimination in Yemen play roles in access to HIV treatment services which are still considered major barriers toward achieving universal access^17^. In addition, the recent conflict in Yemen and economic stagnation have affected people’s lives at all levels and restricted their chance to get access to health services^21^. This cohort study was not comparing different treatment regimens, it was founded to analyse the selected treatment outcomes and the effectiveness of cART in Yemen for cohorts of PLHIV who are being treated during the period 2007 and 2014.

## Methodology

A retrospective descriptive study was conducted among eight cohorts of PLHIV with a total number of 1,703 who have initiated cART and were known to be on treatment 12 months after initiation of antiretroviral therapy during 2007 and 2014 at five public HIV treatment sites in five main governorates as shown in Table (1).

**Table 1:** Cohorts of PLHIV on cART each Year.

Year	Cohorts of PLHIV on cART
2007	104
2008	113
2009	126
2010	288
2011	172
2012	197
2013	270
2014	433
Total	1703

Yemen Cohort analysis has been following PLHIV receiving cART using routine clinical care data where patients follow-up are recorded at ART register. The main outcomes of interest are retention in care, clinical presentations, clinical failures, changes in the cART, lost to follow-up, and mortality.

Excel sheet for cohort analysis is adapted from the WHO cohort report and used to gather data on patients starting cART on zero month, then after 6 months and 12 months. The number of transferred in and transferred out is also gathered. In addition, original cART regimen, substitution, switching, stopped cART, lost to follow-up, death and CD4 are collected as main indicators. Data analysis was done manually by using the excel sheet.

Study limitations are referred to the current insecurity situation and armed conflicts in Yemen that influences on access to more data related to HIV treatment outcomes. Also, this study was subjected to a selection and information bias which can be considered as study limitations in a retrospective study. Only cohorts of PLHIV who were accessed HIV treatment sites at five governorates of Yemen and initiated cART within 2007–2014 have been included in this study.

This research was approved by the Research Ethics Committee at the National University of Malaysia with a reference number: UKM PPI/111/8/JEP-2016-614. And the methods were carried out in accordance with the relevant guidelines and regulations.

## Results

### Socio-demographic characteristics

Table (2) shows 1703 patient profiles were characterized by a predominance of men (male: female ratio 1.6:1 (1039/664)), with a mean age of 35 years and the highest percentage 35% of PLHIV were among the age group of 30–39 year. 85% (1448/1703) had been infected with HIV via heterosexual contact as stated by their notification reports where there may be a bias in self-reporting.

**Table 2:** Characteristic of PLHIV.

Variables	N	%
**Age (Years)**		
0–14	84	5
15–29	411	24
30–39	598	35
40–49	386	23
>49	224	13
**Sex**		
Males	1039	61
Females	664	39
**Exposure category**		
Heterosexual	1448	85
Homosexual	102	6
MTCT	17	1
Blood transfusion	136	8
**CD4**		
≤200	886	52
201–350	530	31
>350	287	17

### Clinical and immunological profiles for PLHIV

**Figure 1:**
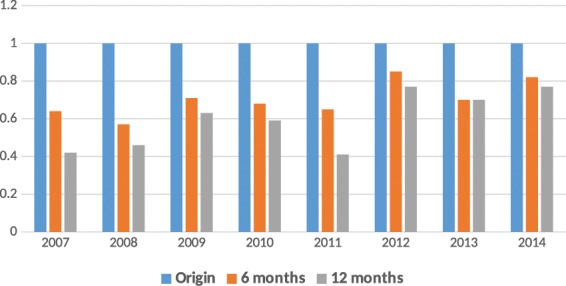
Retention rates of PLHIV Cohorts 6 and 12 months after cART initiation each year during 2007–2014.

**Figure 2:**
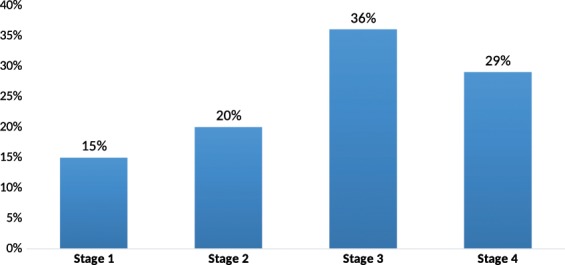
Percentages of PLHIV on WHO Clinical Stages during the first clinic visit through 2007–2014.

Table (2) also reflects that 52% (886/1703) of PLHIV initiated cART with a CD4 T-cell count ≤200 cells/mm3. The cohort analysis of each year during 2007 and 2014 showed 12 months retention rate after cART initiation was less than 80% as shown in Fig. (1). Figure (2) also reflects that 65% (1107/1703) of PLHIV presented late with clinical stages 3 and 4.

### cART regimens used

As it was reported by the year of 2014, Table (3) reflects that 61% (1039/1703) of the cART regimens included Tenofovir and Efavirenz cART. Also, 5% (85/1703) of PLHIV have had initiated TB treatment. 67% (1141/1703) experienced substitution of cART within the first-line regimens, and 22% (375/1703) of PLHIV presented with HIV-related clinical manifestations after a year of cART initiation. However, around 1% (22/1703) of them switched to a second-line cART as shown in Table (3).

**Table 3:** cART Regimens prescribed in Yemen.

First-line cART Regimen	N	%
**cART Regimen**		
Tenofovir (TDF) 300 mg /Emtricitabine (FTC) 200 mg/Efavirenz (EFV) 600 mg	1039	61.01
Zidovudine (AZT) 300 mg/Lamivudine (3TC) 150 mg/Efavirenz (EFV) 600 mg	356	20.90
Tenofovir (TDF) 300 mg /Emtricitabine (FTC) 200 mg/Nevirapine (NVP) 200 mg	175	10.28
AZT 300 mg/3TC 150 mg/NVP 200 mg	91	5.34
Abacavir (ABC) 300 mg/3TC 150 mg/NVP 200 mg	11	0.65
ABC 300 mg /3TC 150 mg/EFV 600 mg	9	0.53
**Second – line Regimen**		
TDF 300 mg/FTC 200 mg/Lopinavir/ritonavir (LPV/r) 400 mg/100 mg	5	0.29
AZT 300 mg/3TC 150 mg/LPV/r 400 mg/100 mg	10	0.59
ABC 300 mg/Didanosine (ddI) 400 mg/LPV/r 400 mg/100 mg	7	0.41
Total	1703	100

### Treatment outcomes

Since the start of the HIV treatment program, there has been an increasing number of PLHIV who access cART each year, with 107 PLHIV in 2007 compared to 433 in 2014 as shown in Table (1). Meanwhile, there was an increased number of AIDS-related death and LTFU. The mean AIDS mortality rate was 15%, while the mean LTFU rate was 16% for the period 2007–2014.

## Discussion

This is the first cohort report analysis conducted in Yemen to highlight selected treatment outcomes. HIV-Yemen Cohort Analysis showed PLHIV in Yemen have been exposed to a wide array of cART regimens within first- and second-line cART. The socio-demographic characteristic of PLHIV is similar to that reported by other countries that are male dominant and have a mean age of around 35 years old. The socio-cultural barriers in Yemen prevent women from seeking medical advice, making them less likely to be tested for HIV. In the Brazil cohort study, the mean age was reported as 36.9 years with a male-to-female ratio of 1.7-1^22^. Meanwhile, in a study by the British Columbia Centre for Excellence in HIV/AIDS which investigated treatment outcomes among those who initiated cART at high CD4 cell count, 80% of study participants were male. Similarly, male represent 71.6% of PLHIV in the study of cART and viral suppression among immigrants in the US^23^. The exposure category of the heterosexual is considered the main mode of HIV transmission in Yemen as seen in most countries. The study of transmitted drug resistance and cART outcomes in South East Asia indicated that heterosexual contact is the main exposure category among the study population^24^.

The majority of patients in this cohort analysis have clinical stages 3 and 4 at the time of cART initiation which is similarly noticed in Mozambique cohort study where clinical stages 3 and 4 represented the highest percentage (24). Also, CD4 count at the time of cART initiation among PLHIV less than 200 cell/mm3 as seen in the study of the association between food insufficiency and HIV treatment outcomes in New York where they found a correlation between low CD4 count and food insufficiency^25^. There is a significant association between a CD4 cell count at baseline and immune outcome; a late initiation of cART with a low CD4 at baseline leads to poor immune recovery^26^. This is also observed in this analysis where most of PLHIV initiated cART at a low CD4 cell count.

The most frequent regimen used in Yemen and worldwide consist of a backbone of Tenofovir (TDF) and Efavirenz (EFV). This analysis showed the highest percentage of PLHIV 61% used TDF/3TC/EFV. However, 22% of PLHIV presented with HIV-related clinical manifestations one year after they initiated cART. More than half of PLHIV 67% received cART substitution due to delayed or inadequate ARV supply at treatment sites. Second-line regimens were also initiated for a small number of PLHIV as evident in this analysis. The inadequate forecasting of second-line cART and lack of stock may be due to a lack of assessment for early warning indicators that should be done at treatment sites and the absence of VL test. Similarly, 47% of Malawi HIV-infected women used TDF/3TC/EFV^27^. It is also recognized that 85% of first-line regimens in Asia have included the same cART^28^. However, treatment failure is considered with frequent treatment substitution where about 20% of PLHIV fail first-line non-nucleoside reverse transcriptase inhibitor within the first few years after cART initiation^29^. In Europe, around 20% of those who initiated cART in chronic HIV infection with undetectable VL are exposed to relapse by the 24th month after the initial virologic response. The relapse rate was high in men, IDUs, those treated in earlier years and those re-starting treatment with very low VL, which may be due to poor adherence to treatment^30^.

TB and HIV comorbidity is considered one of the serious worldwide public health challenges, especially in the European region. HIV infection increases the risk of reactivation of latent TB, and TB has an adverse effect on PLHIV where the disease increases viral replication^31,32^. South Africa has a high burden of HIV/TB co-infection where 61% of TB cases in 2014 have HIV^33^. In Kenya, TB/HIV represented 3.4% of total TB cases^19^, and most European Countries reported 7.9% TB/HIV co-infection of total TB cases^32^. Although the estimated number of TB cases per year in Yemen is 14000, the study among TB patients that was conducted in 2009 showed the prevalence of HIV among TB patients 1.75%^8^. However, this analysis pointed out TB treatment has started for only 5% of PLHIV with proven TB diagnosis in Yemen.

This study pointed out that around 16% of PLHIV are LTFU each year. Similarly, Malawi and Kenya experienced LTFU among PLHIV. The PLHIV who are living in Malawi where the LTFU rate was 17.1% in the first 6 months after cART initiation^27^. In Kenya, the LTFU reached 32.2% within the first 12 months of cART initiation especially among the age group of 20–24 years^19^. It is also estimated that patient retention rates in Africa 3 years after cART initiation is low as 65% due to high LTFU^3^.

Since launching the HIV treatment program in Yemen, AIDS-related deaths have continued to increase although the increasing number of PLHIV who enrolled in HIV care each year due to limited access to cART, high rate of LTFU and poor treatment adherence. The mean AIDS mortality was 15%. In comparison, in other African countries like Malawi and Kenya, the death rates were 0.7% and 3.9%, respectively, within the first 24 months of cART initiation^19,27^. Also, the analysis conducted in West Africa showed that there were 12.1% deaths during the observation period of the study^34^.

HIV related stigma and discrimination have been associated with a high risk of sexual behaviour and low uptake of HIV testing and counseling. PLHIV also admitted that emotional distress due to stigma led to poor cART adherence and decreased likelihood of disclosing their HIV status^35,36^. Also, stigma represents a primary driver of poor HIV outcomes among migrants in high-income countries^37^. In Yemen, stigma and the limited number of HIV treatment sites posed as challenges in access to services^7^.

## Conclusion

This cohort study is conducted for the first time in Yemen and can be used as a baseline guide for future studies. This facilitates analysis of the long term impact of using cART in the context of the routine care of PLHIV at HIV treatment sites in Yemen. Access to cART is low among the female and young age group. An in-depth study should be conducted in the future to identify which at risk groups have low access to cART. Low retention rate, late presentation and low CD4 count during cART initiation have negative treatment outcomes. Switching to a second-line regimen is rare in Yemen due to lack of clinical and laboratory evaluation and lack of adequate stock of second-line ARVs which should be taken into account in the future. TB screening among PLHIV can help to improve HIV treatment outcomes, but low coverage of TB treatment among PLHIV influences on HIV treatment outcomes. Also, low access to treatment sites due to stigma and discrimination play roles in treatment adherence. This analysis will help in the evaluation of an HIV treatment program and further improvement of national health policies to meet the needs of PLHIV in a resource-limited situation like Yemen through scaling up of HIV treatment services, capacity building of health care providers and improving tracking system for those who LTFU. Continuous monitoring of early warning indicators may play a critical role in helping forecast the need, availability, and accessibility for second-line therapy. Planned interventions and improve counseling are also needed to improve access of females and young people to cART and improve retention in care. Community mobilization may ensure earlier initiation of cART and reduce LTFU.

### Ethics approval and consent to participate

This study was part of a study that undergone to Research Ethics Committee, The National University of Malaysia with a reference number UKM PPI/111/8/JEP-2016–614. All procedures performed in studies were in accordance with the ethical standards of the institutional research committee. All interviewees participated on a voluntary basis, after signing an informed consent. The right to refuse to participate or withdraw from the survey, anonymity and confidentiality were guaranteed, as was data protection.

## Supplementary information

Supplementary Dataset 1

Supplementary Dataset 2

Supplementary Dataset 3

Supplementary Dataset 4

Supplementary Dataset 5

## Data Availability

See ‘Availability of materials and data’ section for more information.
